# Chromosomal anomalies at 1q, 3, 16q, and mutations of *SIX1* and *DROSHA* genes underlie Wilms tumor recurrences

**DOI:** 10.18632/oncotarget.6950

**Published:** 2016-01-20

**Authors:** Filippo Spreafico, Sara Ciceri, Beatrice Gamba, Federica Torri, Monica Terenziani, Paola Collini, Fabio Macciardi, Paolo Radice, Daniela Perotti

**Affiliations:** ^1^ Pediatric Unit, Department of Hematology and Pediatric Onco-Hematology, Fondazione IRCCS Istituto Nazionale dei Tumori, Milan, Italy; ^2^ Molecular Bases of Genetic Risk and Genetic Testing Unit, Department of Preventive and Predictive Medicine, Fondazione IRCCS Istituto Nazionale dei Tumori, Milan, Italy; ^3^ Genomics and Bioinformatics Unit, University of Milan–Fondazione Filarete, Milan, Italy; ^4^ Department of Pathology and Laboratory Medicine Fondazione IRCCS Istituto Nazionale dei Tumori, Milan, Italy; ^5^ Department of Psychiatry and Human Behavior, School of Medicine, University of California, Irvine, CA, USA

**Keywords:** Wilms tumor, recurrence, chromosomal anomalies, SIX1, miRNA processor genes

## Abstract

Approximately half of children suffering from recurrent Wilms tumor (WT) develop resistance to salvage therapies. Hence the importance to disclose events driving tumor progression/recurrence. Future therapeutic trials, conducted in the setting of relapsing patients, will need to prioritize targets present in the recurrent lesions. Different studies identified primary tumor-specific signatures associated with poor prognosis. However, given the difficulty in recruiting specimens from recurrent WTs, little work has been done to compare the molecular profile of paired primary/recurrent diseases. We studied the genomic profile of a cohort of eight pairs of primary/recurrent WTs through whole-genome SNP arrays, and investigated known WT-associated genes, including *SIX1*, *SIX2* and micro RNA processor genes, whose mutations have been recently proposed as associated with worse outcome. Through this approach, we sought to uncover anomalies characterizing tumor recurrence, either acquired *de novo* or already present in the primary disease, and to investigate whether they overlapped with known molecular prognostic signatures.

Among the aberrations that we disclosed as potentially acquired *de novo* in recurrences, some had been already recognized in primary tumors as associated with a higher risk of relapse. These included allelic imbalances of chromosome 1q and of chromosome 3, and CN losses on chromosome 16q. In addition, we found that *SIX1* and *DROSHA* mutations can be heterogeneous events (both spatially and temporally) within primary tumors, and that their co-occurrence might be positively selected in the progression to recurrent disease. Overall, these results provide new insights into genomic and genetic events underlying WT progression/recurrence.

## INTRODUCTION

Thanks to an effective integration of surgery, chemotherapy and, in selected cases, radiotherapy, the overall survival rate for patients affected with Wilms tumor (WT), the most frequent pediatric renal tumor, now exceeds 90%. However, only approximately half of children who suffer from tumor relapse reach second durable remission. Aiming at tailoring therapeutic intensification at relapse, an international consensus has recognized three post-relapse risk groups according to the initial treatment received (which in turn is largely dictated by tumor stage and histology) [1]. However, the clinical behavior within the different risk subgroups has yet to be established, since the probability of response to conventional therapies at recurrence is extremely variable [2]. Therefore, the understanding of molecular features underlying recurrent tumors could help to develop effective treatments. Noteworthy, the next generation of early-phase clinical trials, initially conducted in the context of resistant/recurrent tumors, will probably rely on molecular-targeted drugs.

Different studies, aimed at characterizing the molecular genetics of WTs that are likely to relapse, identified some anomalies possibly associated with an adverse outcome, mainly loss of heterozygosity (LOH) at chromosomes 1p and/or 16q [3–5], copy number (CN) gain at chromosome 1q [6–9], and allelic imbalances at chromosome arms 1q, 3p, 3q, and 14q [8].

WT is genetically heterogeneous, and different genes are associated with its development, among which *WT1* at 11p13, the *WT2* locus at 11p15.5, *WTX* on chromosome Xq11.2 and *CTNNB1* on 3p22.1 (reviewed in Huff [10]). Mutations of *TP53*, mapped to 17p13.1, are associated with anaplastic histology [11].

Recently, mutations affecting the *SIX1* and *SIX2* genes and miRNA processor genes (miRNAPGs), including *DICER1*, *DROSHA* and *DGCR8*, have been reported in WTs [12, 13]. *SIX1/2* and *DROSHA*/*DGCR8* mutations underlie high-risk blastemal-type WTs in patients pre-operatively treated according to the protocols of the Sociètè International d'Oncologie Pediatrique (SIOP). In addition, the co-occurrence of *SIX1/2* and *DROSHA*/*DGCR8* mutations resulted in worse outcome in favorable histology WTs in patients receiving primary nephrectomy according to the protocols of the Children's Oncology Group (COG) [12, 13]. Hence, anomalies affecting these novel WT genes seem to be potentially associated with poor prognosis in WT.

The extreme difficulty in recruiting paired primary/recurrent WT samples of the same patient has so far precluded comprehensive studies comparing their genetic profiles. Only one study previously focused on the genomic evolution that leads to tumor recurrence [14]. From a comparison of 28 primary relapsing WTs and 10 unpaired recurrences, CN gains at a series of non contiguous clones spanning more than 80 Mb from region 1q23.3 to 1q44, and CN losses at 17p were statistically more frequent in recurrent tumors. Comparison of 10 paired tumors at diagnosis and relapse demonstrated CN gains at 5p, 8p12, 15q, 16p and 20q and CN losses of 11q and 17p as events acquired *de novo* in two recurrent tumors [14].

To identify genetic and genomic anomalies acquired and/or shared by recurrences, we performed genomic profiling by whole-genome single nucleotide polymorphism (SNP) arrays with an average resolution of 8 Kb, and characterized alterations affecting WT-associated genes, in nine primary tumors from eight patients (including a bilateral case) and in their respective eight recurrences. Through this approach, we identified common features possibly underlying the evolution that leads a primary disease to recur.

## RESULTS

### Chromosomal regions involved in CN anomalies (CNAs) and allelic ratio (AR) anomalies

**Figure 1:**
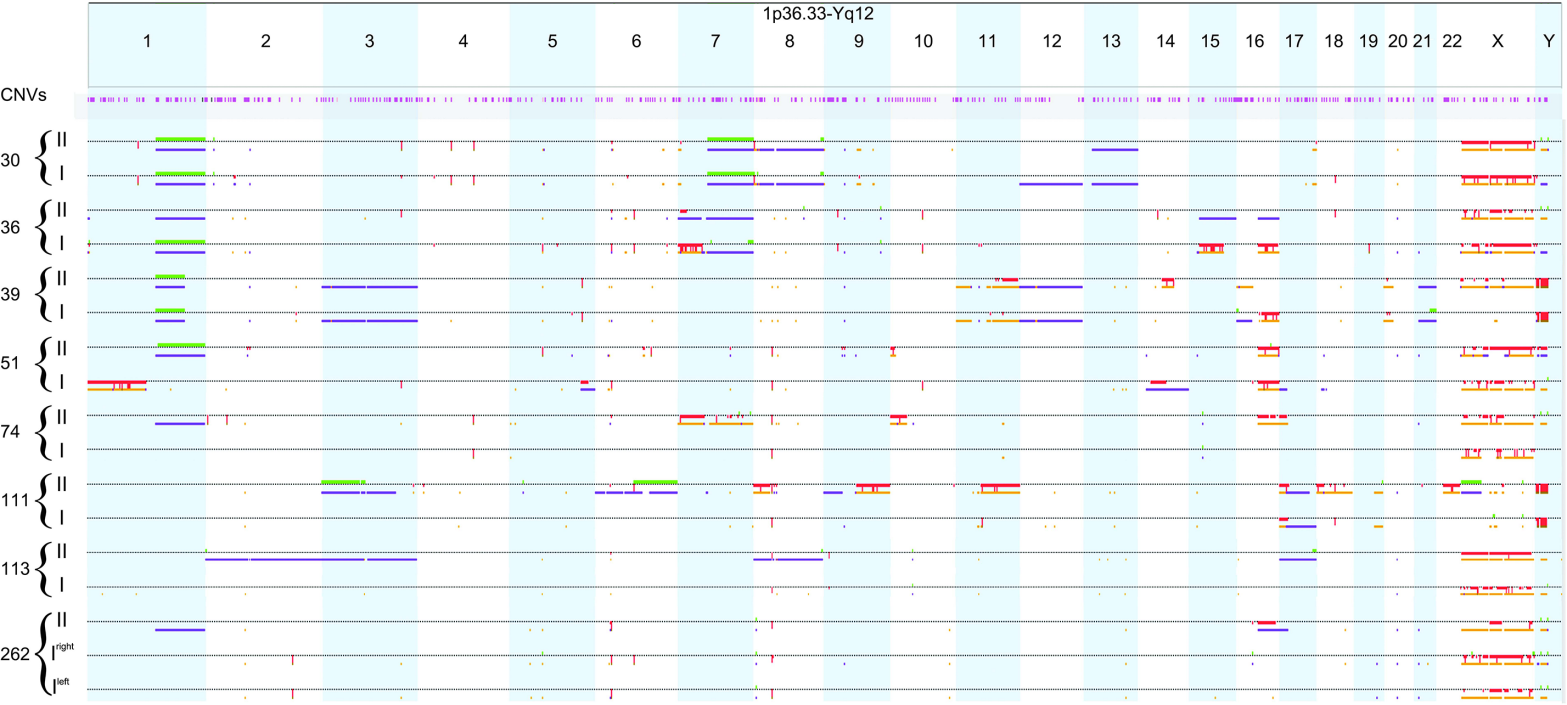
Whole-genome copy number and allelic ratio anomalies in the primary diseases and in their respective paired recurrences Along the genome, data for CN aberrations as well as AR events are displayed for each sample individually on separate rows. CN aberrations are displayed above the dotted line: CN gains are represented by green marks, CN losses by red marks. Allelic events are displayed below the dotted line: allelic imbalances by purple marks and LOH by dark yellow marks. Regions involved in homozygous copy loss are indicated by thicker red marks. The position of constitutional CNVs is indicated in pink. I: primary tumor; II: recurrent tumor.

Whole-genome chromosomal anomalies were examined by SNP array analysis in a set of paired primary tumors and corresponding recurrences (Figure 1). Out of four patients with pre-treated primary tumors, two (36I and 39I) showed multiple gross anomalies involving entire chromosomes/chromosome arms, including 1q CN gain and allelic imbalance and 16q CN loss and LOH, and three tumors from the two remaining cases (74I and 262I, both right and left side) only small focal anomalies (Figure 1). Out of four patients treated with initial nephrectomy at diagnosis, three (30I, 51I and 111I) displayed multiple gross chromosomal anomalies, including 1q CN gain and allelic imbalance (30I), 1p and 16q CN loss and LOH (51I), and one (113I) had only small focal anomalies. Recurrent tumor specimens had aberrations involving entire chromosomes/chromosome arms in all the patients.

Several genetic events, both CNAs and AR anomalies, were observed in regions where constitutional CN variations (CNVs) are located (http://dgv.tcag.ca/dgv/app/home). Among the focal aberrations detected, case no. 30 displayed a focal *MYCN* amplification (chr2:15,495,567-16,243,938) both in the primary tumor (previously reported [8]) and in the recurrence (Figure 1).

### Chromosomal regions involved in CNAs and AR anomalies acquired *de novo* in recurrences

We considered an anomaly as acquired *de novo* in recurrences when in the same chromosomal region the primary tumor had no aberrations or an aberration of different nature. Supplementary Table S1 reports the list of CNAs and AR anomalies that were acquired *de novo* in two or more recurrences. Allelic imbalance of chromosome 1q21.1-q44 was the only anomaly acquired by three recurrences. CN losses of chromosomes 7p22.1, 10p15.3-p14, 11q14.1, 11q14.3, 11q22.1-q25, 16q11.2-q22.1, 16q22.2-q23.3, allelic imbalances of chromosomes 3p26.3-p11.2, 3q11.2-q25.2, 6p22.1-p21.33, 6q24.3, 8p11.21-p11.1, 8q11.1-q11.21, 8q22.3, 16q11.2-q24.3, 17p13.3-p11.2, and LOH of chromosome 10p15.3-p14 were acquired by two recurrent tumors. Some of these anomalies clustered at specific chromosomal arms and encompassed large segments, while others were focal, scattered along the genome and often overlapped with constitutional CNVs (Supplementary Table S1), and therefore might represent spurious observations.

### Chromosomal regions involved in CNAs and AR anomalies shared among recurrences

Supplementary Table S2 reports CNAs and AR anomalies that were present in at least three recurrent tumors. CN gains of chromosome 1q21.2-q32.1 and CN losses of chromosomes 16q11.2-q22.1 and 16q22.2-q23.3 were CNAs commonly present in recurrences. AR abnormalities included allelic imbalances involving chromosomes 1q21.1-q44, 3p26.3-p11.2, 3q11.2-q25.2, 8p11.21-p11.1, 8q11.1-q11.21, 8q22.3, 17p12-p11.2, and LOH of chromosomes 8q11.21-q11.22, 11q22.1, 13q31.2. Again, we could distinguish between anomalies involving large chromosomal segments, and focal, scattered events. Anomalies overlapping with constitutional CNVs are reported in Supplementary Table S2.

### Comparison of chromosomal regions involved in CNAs and AR anomalies acquired *de novo* in recurrences with those shared among recurrences

We compared chromosomal regions involved in anomalies possibly acquired *de novo* in recurrences with those shared among them, and found that CN losses of chromosomes 16q11.2-q22.1, 16q22.2-q23.3, allelic imbalances of chromosomes 1q21.1-q44, 3p26.3-p11.2, 3q11.2-q25.2, 17p12-p11.2, together with a number of focal scattered anomalies were both acquired *de novo* in more than one recurrence and shared by at least three recurrences (Supplementary Table S3).

### *WT1*, *CTNNB1*, *WTX*, *TP53* genetic and genomic anomalies

Sequence analysis disclosed no *WT1* mutations in primary and recurrent tumors. However, SNP array analysis detected anomalies involving the 11p chromosomal region, where *WT1* is mapped, in one case, no. 39 (I and II), that showed CN neutral LOH. No *WTX* gene mutations were identified in all tumor samples, but in a male patient, case no. 30 (I and II), a focal region of deletion on chromosome X, encompassing the *WTX* locus and thus deleting the only present allele, was found. Among female patients, case no. 111 (I and II) displayed a ca. 6.3 Mb region of CN neutral LOH affecting the *WTX* gene and the recurrent tumor 39II showed CN neutral LOH involving most of the X chromosome.

A heterozygous *CTNNB1* c.133T>C, (p.S45P), mutation was found in one case, no. 36 (I and II). Chromosomal anomalies affecting the entire 3p region, where the *CTNNB1* gene is mapped, were found in case no. 39 (I and II) and in the recurrence 113II, which showed allelic imbalance, and in recurrence 111II, which showed CN gain and allelic imbalance.

In one case, no. 111, both primary and recurrent tumors showed CN loss and LOH involving the 17p chromosomal region, where the *TP53* gene is mapped. Although histologically this patient was described as showing diffuse anaplasia both in primary and recurrent disease, we identified a hemizygous *TP53* c.817C>T (p.R273C) mutation only in the recurrence. This was possibly due to the absence of anaplastic cells in the primary tumor specimen that was used for DNA extraction.

Chromosomal anomalies affecting the 17p region were also identified in the recurrence 74II, which showed CN loss and LOH, in the primary tumor 51I, and in the recurrences 113II and 262II, which showed allelic imbalances.

### *SIX1, SIX2*, and miRNAPGs genetic and genomic anomalies

The previously reported *SIX1* hotspot c.530A>G (p.Q177R) mutation was found in heterozygosity in three recurrences: 51II, 74II and 262II. In these samples, cDNA sequencing demonstrated expression of both the mutated and wild-type alleles. While in two of the corresponding primary samples (51I and 262, both right and left side) we excluded the presence of the mutation, genomic sequencing of tumor no. 74I showed the subclonal presence of the mutated allele and cDNA sequencing showed the expression of both the wild-type and mutated alleles (Supplementary Figure S1). All *SIX1* identified mutations were somatic. Chromosomal anomalies affecting the 14q region, where the *SIX1* gene is mapped, were found in tumor no. 51I, which showed allelic imbalance, and in case no. 39II, which showed CN loss and LOH. No *SIX2* mutations were found, and only one recurrence, 113II, showed allelic imbalance at 2p, where the *SIX2* gene is mapped. *DROSHA* heterozygous mutations were found in case no. 51, where the c.3451G>C (p.D1151H) was present in both the primary tumor and in the recurrence, in recurrence 74II, bearing the c.3559C>A (p.Q1187K) mutation, and in recurrence 262II, showing the c.3452A>G (p.D1151G) mutation. All mutations, initially identified through cDNA sequencing, were eventually confirmed at genomic level. Primary tumors of cases no. 74I and 262I (both left and right side) showed no *DROSHA* mutation (Supplementary Figure S2). All *DROSHA* identified mutations were somatic. No chromosomal anomalies affecting the 5p13.3 region, where *DROSHA* is mapped, were found. No mutations affecting *DICER1* and *DGCR8* were found, but a rare germline variant, rs202208301 (allelic frequency in NHLBI Exome Sequencing Project (2) = 1/13006), was observed in case no. 36. Allelic imbalance for the 14q chromosomal region, in which *DICER1* is mapped, was found in primary tumor no. 51I, and CN loss and LOH affecting the 22q *DGCR8* chromosomal region in recurrence 111II.

The finding that *SIX1* and *DROSHA* mutations were mainly identified in recurrences prompted us to expand the analysis on primary tumors using DNA from multiple blocks of formalin fixed paraffin embedded (FFPE) specimens and, when possible, from different histological components. While in cases no. 51I and no. 262I (both right and left side tumors) no *SIX1* mutation was detectable in the DNA from any of the different blocks from the primary tumors investigated, case no. 74I showed *SIX1* mutation in all the specimens. *DROSHA* mutations were identified in the DNA of one of six blocks of case no. 51I, in the DNA of two of three blocks of case no. 74I, and in the DNA of two of four specimens from the right side tumor of case no. 262I, but in none of the four specimens from the left side tumor of the same patient (Supplementary Figures S1 and S2).

**Figure 2:**
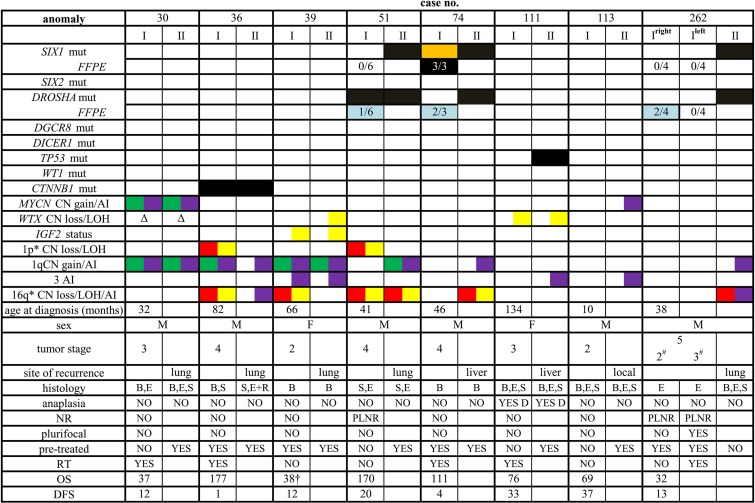
Clinical, pathological and molecular features of the patients investigated I: primary tumor; II: recurrent tumor (“site of recurrence” indicates the site from which sample II was taken), mut: mutation, the presence of somatic mutation is indicated by a black box, by an orange box when subclonal, and by a light blue box when present only in a few of the investigated specimens (indicated by numbers). Chromosomal anomalies are reported as follows: green, CN gain; red, CN loss; yellow, LOH; purple, allelic imbalance (AI), *anomalies < 1 Mb have not been reported. Δ, deletion of the only present allele. All data but those in the line with “FFPE” (formalin fixed paraffin embedded) tag refer to results obtained on frozen material. M, male; F, female; ^#^local stage; B, blastemal component; E, epithelial component; S, stromal component; R, rhabdomyoblastic elements; D, diffuse anaplasia; NR, nephrogenic rests; PLNR, perilobar nephrogenic rests; RT, radiotherapy; OS, overall survival; DFS, disease-free survival; ^†^dead of disease.

Figure 2 summarizes the clinical and molecular characteristics of the patients and of their tumors, including the occurrence of alterations in the investigated WT-associated genes, as well as the presence/absence of the foremost proposed molecular prognostic signatures.

## DISCUSSION

In the present study we describe the findings observed in a cohort of eight pairs of primary/recurrent WTs analyzed by whole-genome SNP array, and we investigated the role in tumor progression of a group of genes, previously reported to be associated with poor outcome in this malignancy [12, 13]. We initially focused on genomic anomalies acquired *de novo* by at least two recurrences. Eventually, we selected aberrations present in at least three recurrent samples (shared events), irrespective of whether they were *de novo* or already present in the primary tumor. In fact, we argued that, while in some cases newly acquired anomalies, not present in the primary disease, are needed for the development of recurrences, in others the recurrence may originate from primary tumors that have already accumulated particularly aggressive molecular characteristics. Finally, in order to identify the possibly more relevant aberrations in recurrent disease, we selected anomalies in common between “*de novo*” and “shared” events.

Chromosomal events acquired *de novo* by more than one recurrence were CN losses at chromosomes 10p15.3-p14, 11q22.1-q25, 16q11.2-q23.3, allelic imbalances at chromosome 1q21.1-q44, at chromosome 3, 16q11.2-q24.3, 17p13.3-p11.2 and LOH at 10p15.3-p14, plus a number of small focal anomalies. Allelic imbalances at chromosomes 1q and 3 were previously identified with higher frequency in relapsing compared to non relapsing primary WTs [8]. The identified CN losses and allelic imbalances at 16q overlap with the small scattered regions that we found as associated with relapse and with prognostically significant chromosomal regions reported by others [3, 4, 8]. Although the different technical approach makes it difficult a comparison with previously published data on paired primary/recurrent samples, CN loss at chromosome 11q is the only anomaly identified as *de novo* event in at least two recurrences in both our and previous study [14]. CN loss and LOH at 10p15.3-p14 and allelic imbalance at 17p13.3-p11.2 represent newly reported *de novo* anomalies acquired by at least two recurrences. However, it must be noted that, since our SNP array-based analysis was performed on a single specimen from each tumor, our characterization of the repertoire of genetic aberrations present in the primary tumor mass might have been incomplete, because of tumor heterogeneity. This makes difficult to state with certainty that those events classified as acquired *de novo* were not already present in a different area of the primary tumor. Furthermore, in sample 36II, tumor cells were intermingled with normal lung cells, leading to a reduction in the representation of the genetic events of tumor cells.

Chromosomal events shared by three or more recurrent tumors were CN gain at chromosome 1q21.2-q32.1, CN losses at 16q11.2-q23.3, allelic imbalances at chromosome 1q21.1-q44, chromosome 3 and chromosome 17p12-p11.2, together with a number of small focal anomalies. Anomalies in common between “*de novo*” and “shared” events included CN losses at chromosome 16q11.2-q23.3, allelic imbalances at chromosome 1q21.1-1q44, at chromosome 3 and chromosome 17p12-p11.2, and focal scattered anomalies, many of which representing CNVs.

The sequence analysis of a group of well established WT-related genes, including *WT1*, *CTNNB1* and *WTX*, was not suggestive of any of these genes being involved in disease progression. *TP53* mutation in recurrence 111II was related to the presence of anaplasia [11], which is considered to be associated with tumor resistance to therapy [15]. The *SIX1* p.Q177R hotspot mutation was found in three out of the eight recurrences: in two cases it appeared to be acquired *de novo*, whereas in one it was already present in all the three different FFPE blocks investigated from the primary tumor and as a subclonal anomaly in the frozen primary tumor specimen. Mutations affecting the RNAse IIIB domain of *DROSHA* were identified in the same three recurrences: two of them derived from primary tumors displaying the presence of perilobar nephrogenic rests (PLNRs), in keeping with previous data [12]. In all the three cases, the mutation was already present in at least one of the DNA samples from multiple areas of the corresponding primary tumors, but never in all samples. Interestingly, primary tumor no. 74I, the only one in which some areas presented the co-occurrence of *SIX1* and *DROSHA* mutations, was a pre-treated blastemal-type WT. Overall, our data suggest that in a fraction of relapsing WT patients *SIX1* and *DROSHA* mutations might be (whether spatially or temporally) heterogeneous events within the primary tumor, which are positively selected in the process of tumor progression to recurrence. In addition, our findings provide clues on the molecular mechanism explaining the association of the above alterations with poor prognosis.

*SIX1*, *SIX2* and miRNAPGs have been reported to be involved in regulating renal development [16–18]. This is consistent with the recent report of the occurrence of mutations of these genes in a fraction of WTs, a malignancy that develops as a consequence of a derangement of kidney embryogenesis [12, 13, 19]. In the latter studies, these mutations were observed at relatively high allelic fractions, suggesting that they represent early events in tumor development. Our data add to this scenario the possibility that alterations of the *SIX1* and *DROSHA* genes occur also at later stage, driving tumor evolution toward chemotherapy resistance and recurrence.

In conclusion, we identified genomic and genetic events, previously associated with poor prognosis in primary relapsing WTs, that appear to be present in a significant fraction of recurrent tumors, possibly as *de novo* events in a subset of cases. We believe that these observations are noteworthy, because they confirm the importance of these anomalies in driving tumor progression and recurrence, and provide further evidence that primary tumors already bearing these defects may deserve particular clinical attention. In fact, the characterization of the full spectrum of molecular events involved in the evolution from primary to recurrent tumors might provide new insights into the design and planning of future clinical trials. A major limitation of this study is represented by the small number of cases analyzed, that might have biased our conclusions. As already anticipated, this is due to the rarity of WT recurrences. Therefore, only the exploitation of ongoing international cooperative initiatives will allow to verify, and possibly confirm on a greater number of paired cases, the observations here reported.

## MATERIALS AND METHODS

### Patients and material

The study included eight patients with WT enrolled into the Associazione Italiana Ematologia Oncologia Pediatrica (AIEOP) protocols TW-2003 (ongoing) and TW-1992 (closed). The treatment protocols had been approved by the participating institutions’ review boards, and specific informed consent to the use of biological samples for the aim of the study obtained from the parents or the guardians of all patients. Eligibility to the study was exclusively based on the availability of matched samples from diagnosis (primary tumor, I) and recurrence (recurrent tumor sample, II). Clinico-pathological characteristics and survival data are depicted in Figure 2. All patients had sporadic tumors, and without signs of any syndromic condition predisposing to WT development.

DNA and RNA were extracted from frozen tumor tissue fragments sampled by a pathologist, using conventional methods. A detailed histology of the frozen tumor specimen used in the study is not available. For selected cases, multiple blocks of FFPE material of the primary tumor mass/masses were checked and macro dissected (> 95% viable tumor cells) to investigate more exhaustively the primary neoplasm and its different histological components. In particular, six different blocks were investigated for tumor no. 51I, corresponding to the blastemal component (four blocks), stromal component with rhabdomyoblastic differentiation (one block), epithelial component with papillary features (one block). Three different blocks with blastemal component were investigated for tumor no. 74I. For the tumor no. 262I of the right side, three different blocks were investigated: one block with epithelial component with papillary features, one block with epithelial component with tubular structures, and one block from which two different areas were macro dissected, one with epithelial component with glomerular structures, and one with epithelial component with glomerular structures and stromal component with rhabdomyoblastic differentiation. For the tumor no. 262I of the left side, three different blocks were investigated: two blocks with epithelial component with papillary features and one block from which two different areas were macro dissected, one with epithelial and blastemal component, and one with epithelial component with glomerular structures. For this case, which was plurifocal, there were no further primary tumor masses not investigated. DNA was extracted using the GeneRead^™^ DNA FFPE Kit (Qiagen, Milan, Italy).

### Genotyping and generation of copy number and allelic event calls

DNA from frozen tumor tissue was analyzed using Illumina 370CNV genotyping BeadChip arrays (370 K). The Infinium II Genotyping reaction steps were performed according to manufacturer's specifications (Illumina, San Diego, CA, USA) at the CBM genotyping service, Trieste, Italy. Normalized bead intensity data obtained for each sample were analyzed with Illumina Genome Studio v1.0.2 software using the manufacturer's default cluster settings, which generates SNP genotypes from fluorescent intensities, together with the normalized measure of the total signal intensity for the two alleles of a SNP, the Log R ratio (LRR), and the normalized measure of the allelic intensity ratio of the two alleles, the B allele frequencies (BAF). These values were used to detect CNAs and AR anomalies. The SNPRank Segmentation algorithm within Nexus Copy Number^™^ v5 (Biodiscovery Inc., El Segundo, CA, USA) was then used to process LRR and BAF and to perform the CN and allelic event calls. Low-level CN gain and loss were defined as LRR values varying from 0.25 to 0.6 or from −0.25 to −0.7, respectively, high level CN gain when LRR values were > 0.6 and homozygous copy loss (HCL) when LRR values were < −0.7. Three types of AR anomalies are automatically classified by Nexus on the basis of BAF and of the homozygous frequency threshold: LOH, allelic imbalance and total allelic loss (TAL). We set a homozygous frequency threshold of 95% and called an LOH with BAF values > 0.8 or < 0.2 and an allelic imbalance with BAF values varying from 0.2 to 0.4 or from 0.6 to 0.8. A TAL was called if the probes showed a noncanonical profile with bands corresponding to the different possible genotypes. During analyses, discontinuities < 1 Mb within aberrant regions have been smoothened.

All the annotations and map information were based on the hg18 release of the human genome, and retrieved from the different databases linked to Nexus (e.g. UCSC, Ensembl, miRNA database, Database of Genomic Variants, RepeatMasker). The methods here briefly summarized have been previously reported in detail [8]. Raw data are available from the authors on request.

### *WT1*, *CTNNB1*, *WTX*, *TP53, SIX1, SIX2*, and miRNAPGs sequence analysis

Sanger sequencing was performed on DNA/cDNA from frozen tumor material for the following analyses. The entire coding region of *WT1* and *WTX*, the exons 3, 7, 8 of the *CTNNB1* gene, and exons 4 to 9 of the *TP53* gene were screened for mutations. *SIX1* (CCDS9748) and *SIX2* (CCDS1822) p.Q177R hotspot mutations were investigated on genomic DNA, and in mutated cases, the expression of the mutated and wild-type alleles was assessed by cDNA sequencing. *DROSHA* (CCDS47195) cDNA spanning aminoacids 936–1253, covering RNAse IIIa and RNAse IIIb domains mutations, was sequenced. Identified mutations were confirmed at genomic level sequencing the affected exon. For primary WT 262I of the left side, in which only genomic DNA was available, *DROSHA* exons 23, 24, 29 and 30 (ENST00000511367), covering previously identified mutations, were sequenced. *DICER1* (CCDS9931) cDNA spanning aminoacids 1682–1829, covering all mutations identified in the RNAse IIIb domain, was sequenced. For primary WT 262I of the left side, corresponding region was investigated through DNA sequencing. *DGCR8* (CCDS13773) p.E518K hotspot was screened by genomic DNA sequencing, whereas aminoacids 59–257 were screened through cDNA sequencing. For primary WT 262I of the left side, genomic DNA was amplified and screened (aminoacids 59–240). DNAs obtained from FFPE material from cases 51I, 74I, 262I (both left and right side) were screened for the specific *SIX1* and *DROSHA* mutations identified. Primer sequences and conditions are described in Supplementary Table S4.

## SUPPLEMENTARY MATERIALS FIGURES AND TABLES


