# Supplementary material for: Chromosomal anomalies at 1q, 3, 16q, and mutations of SIX1 and DROSHA genes underlie Wilms tumor recurrences
Source: Oncotarget. 2016 Jan 20;7(8):8908–15. doi: 10.18632/oncotarget.6950 (PMC4891013; doi:10.18632/oncotarget.6950)
Supplement: Supplementary file 1 [file oncotarget-07-8908-s001.pdf]

Chromosomal anomalies at 1q, 3, 16q, and mutations of *SIX1* and *DROSHA* genes underlie Wilms tumor recurrences

Supplementary Materials

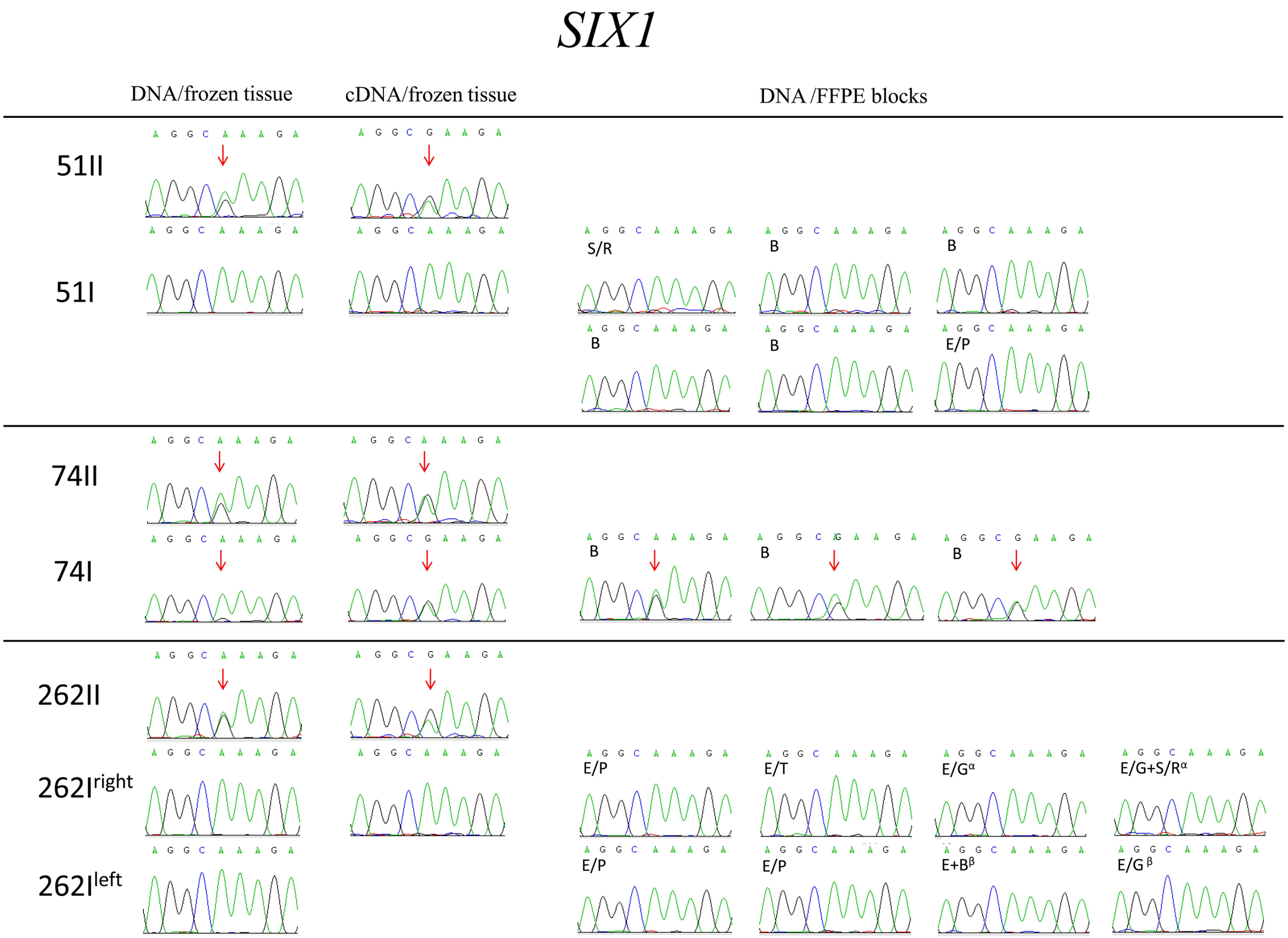

Supplementary Figure S1: *SIX1* mutation screening. Sanger sequencing of genomic DNA and cDNA from frozen primary (I) and recurrent (II) tumor tissue and of DNA obtained from FFPE primary tumor (I) material. The red arrow indicate the c.530A>G mutation. Histology of FFPE material used for DNA extraction is indicated. S/R, stromal component with rhabdomyoblastic differentiation; B, blastemal component; E/P, epithelial component with papillary features; E/T, epithelial component with tubular structures; E/G, epithelial component with glomerular structures; α, different histological components macro dissected from the same FFPE block; β, different histological components macro dissected from the same FFPE block.

DROSHA

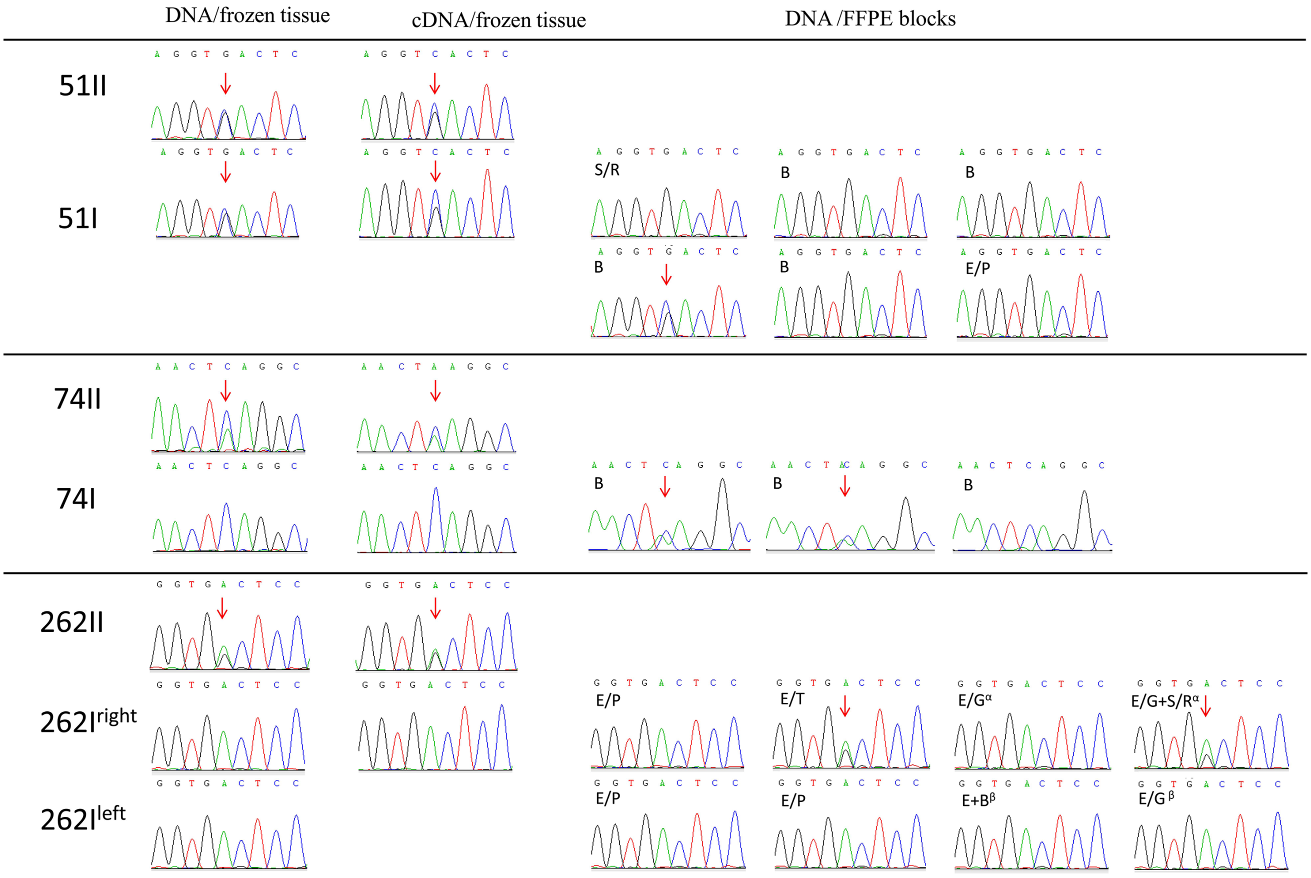

Supplementary Figure S2: *DROSHA* mutation screening. Sanger sequencing of genomic DNA and cDNA from frozen primary (I) and recurrent (II) tumor tissue and of DNA obtained from FFPE primary tumor (I) material. The red arrow indicate the identified mutations. Histology of FFPE material used for DNA extraction is indicated. S/R, stromal component with rhabdomyoblastic differentiation; B, blastemal component; E/P, epithelial component with papillary features; E/T, epithelial component with tubular structures; E/G, epithelial component with glomerular structures; α, different histological components macro dissected from the same FFPE block; β, different histological components macro dissected from the same FFPE block.

Supplementary Table S1: De novo events in recurrences

Chromosome region	Cytoband	Event	Recurrence no.
chr1:142,649,580-247,249,719	q21.1-q44	Allelic imbalance	51, 74, 262
chr2:86,965,048-88,101,382	p11.2 (*)	Allelic imbalance	51, 113
chr3:0-80,020,965	p26.3-p12.3	Allelic imbalance	111, 113
chr3:81,229,116-89,189,181	p12.3-p11.2	Allelic imbalance	111, 113
chr3:94,994,003-155,910,888	q11.2-q25.2	Allelic imbalance	111, 113
chr3:163,998,933-164,105,429	q26.1(*)	CN loss	30, 36
chr3:163,998,933-164,105,429	q26.1(*)	LOH	30, 74
chr6:26,560,982-27,337,036	p22.1	Allelic imbalance	51, 111
chr6:29,686,696-30,168,096	p22.1-p21.33	Allelic imbalance	74, 111
chr6:29,958,544-30,007,366	p21.33(*)	CN loss	111, 262
chr6:32,561,814-32,659,589	p21.32(*)	Allelic imbalance	30, 111
chr6:32,643,909-32,659,589	p21.32(*)	CN loss	30, 74
chr6:147,612,730-148,896,357	q24.3	Allelic imbalance	36, 111
chr7:5,032,254-5,059,656	p22.1	CN loss	30, 74
chr7:61,060,840-62,434,687	q11.1-q11.21(*)	Allelic imbalance	36, 111
chr8:42,314,267-43,951,038	p11.21-p11.1	Allelic imbalance	111, 113
chr8:46,994,719-49,190,162	q11.1-q11.21	Allelic imbalance	111, 113
chr8:103,282,575-103,389,406	q22.3	Allelic imbalance	36, 113
chr9:38,932,768-40,907,166	p13.1-p12(*)	Allelic imbalance	51, 111
chr9:66,194,869-68,225,875	q12(*)	Allelic imbalance	51, 111
chr10:0-9,873,294	p15.3-p14	CN loss	51, 74
chr10:0-9,873,294	p15.3-p14	LOH	51, 74
chr11:83,482,872-84,421,016	q14.1	CN loss	39, 111
chr11:89,562,803-89,587,477	q14.3	CN loss	39, 111
chr11:100,774,878-130,569,344	q22.1-q25	CN loss	39, 111
chr16:44,943,958-88,827,254	q11.2-q24.3	Allelic imbalance	36, 262
chr16:44,943,958-68,540,340	q11.2-q22.1	CN loss	74, 262
chr16:69,720,459-82,457,535	q22.2-q23.3	CN loss	74, 262
chr17:0-19,672,268	p13.3-p11.2	Allelic imbalance	113, 262
chr20:27,100,000-28,255,585	q11.1(*)	Allelic imbalance	51, 111
chr21:9,730,102-10,201,835	p11.2-p11.1(*)	Allelic imbalance	51, 74

De novo events in recurrences: chromosomal regions, cytoband locations and samples involved in the chromosomal events are indicated. Chromosomes X and Y were excluded; (*) indicates a chromosomal region with a percentage of CNV overlap > 80%.

Supplementary Table S2: Shared events in recurrences

Chromosome region	Cytoband	Event	Recurrence no.
chr1:142,649,580-146,391,799	q21.1(*)	Allelic imbalance	30, 36, 39, 51, 74, 262
chr1:146,391,799-147,955,187	q21.1(*)	Allelic imbalance	36, 51, 74, 262
chr1:147,955,187-148,190,820	q21.1-q21.2(*)	Allelic imbalance	36, 39, 51, 74, 262
chr1:148,190,820-204,669,053	q21.2-q32.1	Allelic imbalance	30, 36, 39, 51, 74, 262
chr1:148,190,820-204,669,053	q21.2-q32.1	CN gain	30, 39, 51
chr1:204,669,053-247,249,719	q32.1-q44	Allelic imbalance	30, 36, 51, 74, 262
chr2:82,371,324-83,157,679	p12(*)	LOH	36, 74, 111
chr2:90,970,848-91,680,834	p11.2-p11.1(*)	Allelic imbalance	30, 39, 113
chr3:0-19,337,019	p26.3-p24.3	Allelic imbalance	39, 111, 113
chr3:20,395,567-80,020,965	p24.3-p12.3	Allelic imbalance	39, 111, 113
chr3:81,229,116-89,189,181	p12.3-p11.2	Allelic imbalance	39, 111, 113
chr3:94,994,003-155,910,888	q11.2-q25.2	Allelic imbalance	39, 111, 113
chr5:68,915,847-68,935,325	q13.2(*)	LOH	30, 36, 51, 113
chr5: 68,935,325-69,079,058	q13.2(*)	LOH	30, 36, 39, 51, 113
chr5: 69,079,058-69,699,316	q13.2(*)	LOH	30, 36, 39, 51, 113, 262
chr5: 69,699,316-70,071,849	q13.2(*)	LOH	30, 36, 39, 113, 262
chr5: 70,071,849-70,098,885	q13.2(*)	LOH	30, 36, 39, 262
chr5: 70,098,885-70,238,992	q13.2(*)	LOH	30, 36, 262
chr6:29,958,544-30,017,518	p21.33(*)	Allelic imbalance	74, 111, 262
chr6:32,639,473-32,659,589	p21.32(*)	Allelic imbalance	30, 36, 111
chr6:32,643,909-32,659,589	p21.32(*)	CN loss	30, 36, 74
chr7:61,540,598-63,683,558	q11.21(*)	Allelic imbalance	30, 36, 111
chr7:110,319,308-110,842,066	q31.1(*)	Allelic imbalance	30, 36, 51
chr8:39,353,808-39,503,466	p11.23-p11.22(*)	HCL	51, 74, 111, 113, 262
chr8:39,353,808-39,503,466	p11.23-p11.22(*)	LOH/TAL	39, 51, 74, 111
chr8:42,314,267-43,951,038	p11.21-p11.1	Allelic imbalance	30, 111, 113
chr8:46,994,719-49,190,162	q11.1-q11.21	Allelic imbalance	30, 111, 113
chr8:50,157,278-51,041,715	q11.21-q11.22	LOH	39, 51, 113
chr8:103,282,575-103,389,406	q22.3	Allelic imbalance	30, 36, 113
chr9:43,515,795-46,336,803	p11.2(*)	Allelic imbalance	30, 36, 39, 51
chr11:99,103,230-101,101,789	q22.1	LOH	39, 74, 111
chr13:88,157,194-88,767,293	q31.2	LOH	39, 113, 262
chr16:44,943,958-68,540,340	q11.2-q22.1	CN loss	51, 74, 262
chr16:69,720,459-82,457,535	q22.2-q23.3	CN loss	51, 74, 262
chr17:15,637,203-19,672,268	p12-p11.2	Allelic imbalance	111, 113, 262
chr20:27,100,000-28,255,585	q11.1(*)	Allelic imbalance	36, 51, 111, 113, 262
chr21:9,730,102-10,201,835	p11.2-p11.1(*)	Allelic imbalance	39, 51, 74, 262

Shared events in recurrences: chromosomal regions, cytoband locations and samples involved in the chromosomal events are indicated. Chromosomes X and Y were excluded; (*) indicates a chromosomal region with a percentage of CNV overlap > 80%.

Supplementary Table S3: Common anomalies between de novo and shared events in recurrences

Chromosome region	Cytoband	Event
chr1:142,649,580-247,249,719	q21.1-q44	Allelic imbalance
chr3:0-19,337,019	p26.3-p24.3	Allelic imbalance
chr3:20,395,567-80,020,965	p24.3-p12.3	Allelic imbalance
chr3:81,229,116-89,189,181	p12.3-p11.2	Allelic imbalance
chr3:94,994,003-155,910,888	q11.2-q25.2	Allelic imbalance
chr6:29,958,544-30,017,518	p21.33(*)	Allelic imbalance
chr6:32,639,473-32,659,589	p21.32(*)	Allelic imbalance
chr6:32,643,909-32,659,589	p21.32(*)	CN loss
chr7:61,540,598-62,434,687	q11.21(*)	Allelic imbalance
chr8:42,314,267-43,951,038	p11.21-p11.1	Allelic imbalance
chr8:46,994,719-49,190,162	q11.1-q11.21	Allelic imbalance
chr8:103,282,575-103,389,406	q22.3	Allelic imbalance
chr16:44,943,958-68,540,340	q11.2-q22.1	CN loss
chr16:69,720,459-82,457,535	q22.2-q23.3	CN loss
chr17:15,637,203-19,672,268	p12-p11.2	Allelic imbalance
chr20:27,100,000-28,255,585	q11.1(*)	Allelic imbalance
chr21:9,730,102-10,201,835	p11.2-p11.1(*)	Allelic imbalance

Anomalies in common between "*de novo*" and "shared" events: chromosomal regions and cytoband locations involved in the common chromosomal events are indicated. (*) indicates a chromosomal region with a percentage of CNV overlap > 80%.

Supplementary Table S4: *WT1* primers used for DNA PCR and sequencing

Primer	Sequence	Annealing T (°C)
WT1 1aF	AGCCAGAGCAGCAGGGAGTC	
WT1 1aR	AACGACCCGTAAGCCGAAGC	60
WT1 1bF	CGCCGGTGCTGGACTTTG	
WT1 1bR	CGGTCAAAAGGGGTAGGAGA	60
WT1 2F	CCGTCTTGCGAGAGCACC	
WT1 2R	CTAATTTGCTGTGGGTTAGG	56
WT1 3F	GCTCAGGATCTCGTGTCTCC	
WT1 3R	TCCCAAGGACCCAGACGCAG	60
WT1 4F	TGTGCAGAGATCAGTGGGATG	
WT1 4R	CAGAGAGCTTTGCCCTTTCTTC	60
WT1 5F	CACTGGATTCTGGGATCTG	
WT1 5R	CTGCATTGCCCCAGGTG	60
WT1 6F	CCAAATGGCGACTGTGAGCC	
WT1 6R	CAGGGCCAAAGAGTCCATC	60
WT1 7F	GACCTACGTGAATGTTCACATG	
WT1 7R	CTCTTGAACCATGTTTGCCCAAGAC	54
WT1 8F	AGTCCTAGTAGGAGAGGTTGCC	
WT1 8R	CACATGGCTGACTCTCTCATTC	60
WT1 9F	GAAGTCAGCCTTGTGGGC	
WT1 9R	GCCACGCACTATTCCTTCTCTC	60
WT1 10F	GAGTGGGTGCCTTGTGATGAC	
WT1 10R	TGCCTGGGACACTGAACGG	60

WTX primers used for DNA PCR and sequencing [1]

Primer	Sequence	Annealing T (°C)
WTX aF	CCAGACCCCACTGTGATGCTTCCTG	
WTX aR	CAGCCACAGATGTCTTACATCTGGAGC	70
WTX bF	CAGCTCCAAGAAAGGTCTCAGCAAG	
WTX bR	CTGGATCTTTACAGGCCATTTTCTC	65
WTX cF	GTCAGAGCCAGGCCTCATGAGCACG	
WTX cR	CATCATCATCTGGCAAGGCCATCTC	70
WTX dF	GGGATGTGACATCCCTGAAAAGCTTTG	
WTX dR	CCTCACCTGAATCATCCTCAAATCC	65
WTX eF	CAATATGAACCTGGGCTACCATCCCAC	
WTX eR	CTGGCATGAGCTTCTCGGGCACGTG	70
WTX fF	CCTCCATGGTCGAAGCTCTGAGATG	
WTX fR	CATAACGCTTCTCCAGAGGACGGAAG	70
WTX gF	GAGTATCAGATGAGGCCCTTAGG	
WTX gR	CACATCAGCGATGTCAAAGGTCACC	65
WTX hF	GGAATGCCACTGTGAGTTTCTCACAGG	
WTX hR	GTCCTCCTCATCTGAATCTTCCTGC	68
WTX iF	GTGAGCAGCCTCCCTCGATACTTGG	
WTX iR	ACGAGCTAGTTGAGGCCCAGATTC	68

WTX jF	CCAACCAGTTGGACAGGCCTTCCAG			
WTX jR	CTAGGTTTCCATTCATGGCAGTGGAG			70
WTX kF	CTTCCAGTTCTGGAGGCTTCAGC			
WTX kR	GCAGATGCACTTGAGTTGAACGTGG			65

***CTNNB1* primers used for DNA PCR and sequencing**

Primer	Sequence	Annealing T (°C)
CTNNB1 3F	GCTGATTTGATGGAGTTGGA	
CTNNB1 3R	CCAGCTACTTGTTCTTGAGTGAA	58
CTNNB1 7F	GTTGGTAATATGGCTCTTCTCAGAC	
CTNNB1 7R	GGCTGCAAACTGAATAGGACC	58
CTNNB1 8F	TAGGATTGATAGGCACTTCTAGC	
CTNNB1 8R	AGGAGACCTTCCATCCCTTC	58

***TP53* primers used for DNA PCR and sequencing**

Primer	Sequence	Annealing T (°C)
TP53 4F	GCTGAGGACCTGGTCCTCTGAC	
TP53 4R	GAAGAGGAATCCCAAAGTTCCA	56
TP53 5F	TTTGTTTCTTTGCTGCCGTCTTC	
TP53 5R	AGGCCTGGGGACCCTGGGCA	58
TP53 6F	GCTGGTTGCCCAGGGTCCCCAG	
TP53 6R	CCTGGAGGGCCACTGACAACCA	63
TP53 7F	CTTGCCACAGGTCTCCCCAA	
TP53 7R	GTCAGAGGCAAGCAGAGGCT	58
TP53 8F	TTGGGAGTAGATGGAGCCT	
TP53 8R	AGGCATAACTGCACCCTTGG	58
TP53 9F	GCAGTTATGCCTCAGATTCAC	
TP53 9R	AAGACTTAGTACCTGAAGGGT	50

***SIX1* primers used for DNA PCR and sequencing**

Primer	Sequence			Annealing T (°C)
SIX1 F2	GCGCACCATCTGGGACGG			58
SIX1 R2	GAGGAGAAAGGACGGCTTCC	[2]		
SIX1 F4	TCCCTACCCATCGCCGCGT			
SIX1 R2	GAGGAGAAAGGACGGCTTCC	[2]	FFPE DNA	58

***SIX1* primers used for cDNA PCR and sequencing**

Primer	Sequence		Annealing T (°C)
SIX1 F2	GCGCACCATCTGGGACGG		62
SIX1 R5	GCTTGCCCCCTTCCAGAGGAGA	[2]	

***SIX2* primers used for DNA PCR and sequencing**

Primer	Sequence		Annealing T (°C)
SIX2F	CCCGCGAGAAGCGTGAGC		58
SIX2R	GGGTCTCCGGCATAGAAAGG	[2]	

***DGCR8* primers used for DNA PCR and sequencing**

Primer	Sequence		Annealing T (°C)
DGCR8F	GGGAAATCCGAGGTCTGCAT	[2]	58
DGCR8R	CTTCCCTTTCCTCCCGTTCC	[2]	
DGCR8 2F	GCTCTGGTGGTGATGGACAG		59
DGCR8 2R	GATGCTGCAATTCCCAAATCTCTC		

***DGCR8* primers used for cDNA PCR and sequencing**

Primer	Sequence	Annealing T (°C)
DGCR8 2F	GCTCTGGTGGTGATGGACAG	59
DGCR8 cDNA 2R	CCTCTGTTCGCCTCTTTTTGGG	

***DICER1* primers used for DNA PCR and sequencing**

Primer	Sequence	Annealing T (°C)
DICER1 cDNA F	CAACTACAGATTCAAGAATAAGGC	58
DICER1 R1	CCCTTGAATATTAAGCATGAACAC	
DICER1 F2	GGATTTGGGGATCAGTTGCTATG	60
DICER1 R2	CCTGCTGTCCCTTTAGACCAC	
DICER1 F3	CTACATCTGTGGACTGCCTG	58
DICER1 cDNA R	GGATAGTACACCTGCCAGAC	

***DICER1* primers used for cDNA PCR and sequencing**

Primer	Sequence	Annealing T (°C)
DICER1 cDNA F	CAACTACAGATTCAAGAATAAGGC	56
DICER1 cDNA R	GGATAGTACACCTGCCAGAC	

***DROSHA* primers used for DNA PCR and sequencing**

Primer	Sequence		Annealing T (°C)
DROSHA 23F	CTAAAGGAGCTAACCCTCATGTG		58
DROSHA 23R	GAAAGCAATGTAATAAGTATGTTACC		
DROSHA 24F	CACAGACAGAATGTCGTGATGC		58
DROSHA 24R	CTTATTCAAATGCAAAATTATGCATGG		
DROSHA 29F	CTTAGGGAATTGTATTTGGTTTTGTG		56
DROSHA 29R	ATATTTGACCCCTCTAATCTTCACTC		
DROSHA 30F	GTCCAACCCAGGAATAAGAGAG		58
DROSHA 30R	GAACATAATAATAATGAGGAGGAGGAC		
DROSHA F4	GGTTATTGATTTCTTTACTTTGATG		
DROSHA 29R	ATATTTGACCCCTCTAATCTTCACTC	FFPE DNA	56
DROSHA 30F	GTCCAACCCAGGAATAAGAGAG		60
DROSHA R3	GGCCTCTTGGTCTTGTCGTTG	FFPE DNA	

***DROSHA* primers used for cDNA PCR and sequencing**

Primer	Sequence		Annealing T (°C)
DROSHA cDNA 1F	GGAGACAGAAAAGTTCATCACATG	[3]	56
DROSHA cDNA 1R	GATAATTGAGCCAGACTTCGC	[3]	
DROSHA cDNA 2F	GCGAAGTCTGGCTCAATTATC	[3]	56
DROSHA cDNA 2R	GGGTCATTCCAATCCTGATTC	[3]	

REFERENCES

1. Rivera MN, Kim WJ, Wells J, Driscoll DR, Brannigan BW, Han M, Kim JC, Feinberg AP, Gerald WL, Vargas SO, Chin L, Iafrate AJ, Bell DW, et al. An X chromosome gene, WTX, is commonly inactivated in Wilms tumor. Science. 2007; 315:642–5.
2. Wegert J, Ishaque N, Vardapour R, Geörg C, Gu Z, Bieg M, Ziegler B, Bausenwein S, Nourkami N, Ludwig N, Keller A, Grimm C, Kneitz S, et al. Mutations in the SIX1/2 pathway and the DROSHA/DGCR8 miRNA microprocessor complex underlie high-risk blastemal type Wilms tumors. Cancer Cell. 2015; 27:298–311.
3. Torrezan GT, Ferreira EN, Nakahata AM, Barros BD, Castro MT, Correa BR, Krepischi AC, Olivieri EH, Cunha IW, Tabori U, Grundy PE, Costa CM, de Camargo B, et al. Recurrent somatic mutation in DROSHA induces microRNA profile changes in Wilms tumour. Nat Commun. 2014; 5:4039.
